# Environmental Influences on Mammographic Breast Density in California: A Strategy to Reduce Breast Cancer Risk

**DOI:** 10.3390/ijerph16234731

**Published:** 2019-11-27

**Authors:** Barbara A. Cohn, Mary Beth Terry

**Affiliations:** 1 Child Health and Development Studies, Public Health Institute, Berkeley, CA 94709, USA; 2 Herbert Irving Comprehensive Cancer Center and Mailman School of Public Health, Columbia University, New York, NY 10032, USA; mt146@columbia.edu

**Keywords:** mammographic breast density, breast cancer, environmental chemical exposures, cohort, prevention, California, BI-RADS

## Abstract

State legislation in many U.S. states, including California, mandates informing women if they have dense breasts on screening mammography, meaning over half of their breast tissue is comprised of non-adipose tissue. Breast density is important to interpret screening sensitivity and is an established breast cancer risk factor. Environmental chemical exposures may play an important role in this, especially during key windows of susceptibility for breast development: in utero, during puberty, pregnancy, lactation, and the peri-menopause. There is a paucity of research, however, examining whether environmental chemical exposures are associated with mammographic breast density, and even less is known about environmental exposures during windows of susceptibility. Now, with clinical breast density scoring being reported routinely for mammograms, it is possible to find out, especially in California, where there are large study populations that can link environmental exposures during windows of susceptibility to breast density. Density scores are now available throughout the state through electronic medical records. We can link these with environmental chemical exposures via state-wide monitoring. Studying the effects of environmental exposure on breast density may provide valuable monitoring and etiologic data to inform strategies to reduce breast cancer risk.

## 1. Introduction

Mammographic breast density (MBD) is a measure of the amount of fibroglandular breast tissue compared to overall breast tissue and is an established risk factor for breast cancer, and this association is not just from masking [1]. Both the percent and absolute amount of MBD have been shown to be related to breast cancer risk in both pre and post-menopausal women (for meta-analysis [2]). Higher MBD also reduces the sensitivity of mammography [3]. Spearheaded by the late Nancy Cappello [4], U.S. states have enacted legislation to ensure that women with high MBD are informed by their providers, both because it is an independent risk factor and also because it reduces the sensitivity of mammography [5]. 

Twin studies have long supported the importance of genetics and early life environment on MBD, with concordance in monozygotic twins as high as 60% and concordance in dizygotic twins and siblings as high as 30% [6,7,8]. Even though these findings from twin studies suggest a large percentage of MBD may be related to shared genetics and shared early life environment, MBD has also been consistently shown to be modifiable, particularly by exogenous hormones. For example, in one of the largest randomized controlled trials in women’s health—the Women’s Health Initiative (WHI)—Byrne and colleagues demonstrated that not only was hormone replacement therapy (HRT) initiation with estrogen and progestin associated with increases in MBD, but MBD changes were associated with increased breast cancer risk [7]. In a similar way, treatment with tamoxifen has been shown to reduce MBD, and, in fact, MBD has been suggested to be a biosensor of response to tamoxifen [9]. Other established risk factors for breast cancer, like alcohol consumption, have been less consistently associated with MBD [10].

MBD, in addition to its great potential to play a key role as a biosensor, particularly for hormonal treatments, may also hold the key to understanding the complex role of body mass index (BMI) and breast cancer risk. Specifically, the positive association between higher BMI and postmenopausal breast cancer, coupled with the negative association between higher BMI in adolescence and early adulthood and premenopausal breast cancer, is incredibly consistent across many diverse populations and remains poorly understood [11]. The consistency is also seen in women at a higher risk of breast cancer and across the full spectrum of absolute predicted risk [12]. The negative association for adolescent BMI does not extend to earlier in life, as birthweight remains positively associated with breast cancer risk and we have recently reported a similar positive association between birthweight and MBD [13]. Specifically, birth weight was positively associated with dense area, whereas rapid weight gain in infancy and early childhood to four years were negatively associated with dense area (in the overall cohort and in the sibling subset). These results support that the pattern of BMI with breast cancer risk may also be reflected in breast density prior to menopause [13].

We hypothesize the MBD may also hold the key to understanding the role of environmental exposures and breast cancer risk, particularly as it can be used as a biosensor at the breast tissue level for changes over time, as it has been for HRT and other exogenous hormones. However, even though the literature on environmental chemical and breast cancer risk has generally not been specific to measuring exposures during key windows of susceptibility (WOS) when the breast changes in form and function [14], the literature on environmental exposures during WOS and MBD is even more sparse. Cross sectional studies, not specific to a WOS, have reported associations between higher MBD and airborne metals and polycyclic hydrocarbons [15], other heavy metals like magnesium [16], and selected xenoestrogens including BPA [17]. An exception is one cross-sectional study of PCBs, which did not find an association with higher MBD [18]; if anything, selected PCBs were associated with lower MBD. The challenge with cross-sectional studies outside of a WOS is that they cannot measure change in MBD and, therefore, some of the associations between environmental exposures may be missed if the skew of the study population is to older, postmenopausal women, far from the menopausal transition and after MBD has declined. Only two studies, both by our group, have specifically examined environmental exposures and MBD specific to a WOS. We found that in utero DDT exposure was associated with higher MBD and absolute dense area in women at midlife in women whose mothers had breast cancer [19] and also in women unselected based on family history [20]. Although the literature is sparse, these studies support the potential of using MBD to measure the effect of environmental carcinogens on breast cancer risk. 

MBD can be measured continuously, like we did in our studies [19,20], using a semi-automated method named Cumulus. Although such methods are useful, as they can measure small changes in MBD, they are often not feasible for large-scale studies, particularly for biomonitoring of changes over time. The study by White and colleagues [15] also offers promise that large linkage studies, that use widely available clinical reports and link to EPA National Air Toxics Assessment, may be a robust design to measure the effect of environmental chemicals over time. This study measured MBD in 222,581 women using the Breast Imaging-Reporting and Data System (BI-RADS) and thus supports the use of a clinical score like BI-RADS which is reported in four categories: almost entirely fatty (BI-RADS 1), scattered areas of fibro glandular density (BI-RADS 2), heterogeneously dense (BI-RADS 3), and extremely dense (BI-RADS 4). 

Most states report results if a woman has a BI-RADS 3 or 4, and approximately half of all adult women of screening mammography age do have a BI-RADS score in this range [21]. Thus, not only is monitoring breast density at the state level feasible, evidence supports the ability to link to other sources of environmental monitoring data to test hypotheses and use MBD as a biosensor to the effects of environmental chemicals at the breast tissue level. In this commentary, we outline a research program to provide both etiologic, as well as the possibility for ongoing biomonitoring of the environmental, causes of MBD as a step in finding highest risk communities defined by modifiable environmental exposures and times in the life-course when MBD is most impacted by the environment.

## 2. Approach

### 2.1. Framework to Expand Research on Environmental Exposure and MBD

Identify geographic areas with high MBD, defined as heterogeneously dense (BI-RADS 3), and extremely dense (BI-RADS 4), and link these patterns to modifiable environmental exposures via trans-disciplinary designs that include:

- Geographic Information Systems (GIS) studies to map high MBD hotspots and associated geographic distribution of exposures;
- Prospective epidemiological studies that link MBD to environmental factors during WOS;
- Multiple ‘omics investigations to discover individual pathways to risk;
- Developmental toxicology studies to confirm causation and investigate mechanism.

Engage communities of concern in discovery and create advocacy to reduce deleterious exposures via public policy.

Implement an ongoing biomonitoring project that integrates state-wide environmental data with MBD data.

Methods: It is now feasible to conduct large-scale studies of MBD and environmental exposures across the state of California using mapping and other GIS methods. GIS methods can inform prospective human studies that can be rapidly completed in existing long-term California cohorts that include risk factors during WOS. Human cohorts can now be linked to resources for density scoring, making large-scale research feasible and affordable. Based on human findings, metabolomics and experimental animal studies can identify mechanisms and investigate causality. Trans-disciplinary collaboration with metabolomics and developmental toxicology can be pursued and is now feasible, as we have shown [22].

### 2.2. GIS Studies of Environmental Determinants of MBD

Critical features for successful GIS studies of MBD are in place in California. There has been mandatory reporting of MBD in the state since April of 2013 [23]. Mammography is in frequent use in California, with 74% of women age 40 and over screened as of 2016 [24], although there is variation in regular use of mammography [25]. There is an ongoing mammography registry (National Cancer Institute’s Breast Cancer Surveillance Consortium (BCSC) [26] that includes the San Francisco Mammography Registry, covering Petaluma, San Francisco, Berkeley, Walnut Creek, San Mateo, where two million mammograms are available for 450,000 women and consents have been obtained for research [27]. This registry can be immediately accessed to describe geographic hot spots for high MBD.

California has an extensive database that describes the geographic and historic distribution of the use of pesticides beginning in 1991 [28] and has coverage from National Air Toxics Assessment (NATA) for air toxics [29]. A recent study used the NATA data to link MBD assessed by clinical BI-RADS scores to some air pollution components nationally, demonstrating feasibility [15]. Investment in resources to integrate these different, readily available sources of data may have a profound impact on our understanding of environmental factors and MBD. Such linkage after an upfront investment would be a cost-efficient method for both etiological studies as well as ongoing biomonitoring of trends. 

GIS studies offer the further opportunity to integrate social with environmental risk factors. Many diseases, including breast cancer, differ by social factors either in incidence, age at incidence, mortality or aggression, or severity at the time of diagnosis [30]. Few studies have effectively integrated the role of the physical and chemical environment in these disparities [30]. GIS data is linkable to both environmental chemicals and social factors such as poverty and segregation [4,27,31].

Key hypotheses related to environmental exposures and MBD can be tested by using GIS mapping to large-scale MBD registry data. Specific hypotheses related to WOS and MBD will require appropriate assumptions about lagging effects of environmental exposures based on age at mammogram, and average ages at menopause, pregnancy, and menarche. Ecological associations could be then followed up on with individual cohort data, with individual data available for each WOS.

### 2.3. Prospective Epidemiological Studies of the Environment and MBD

A special feature of California, in addition to its size and diversity of population and place, is the presence of long-term cohorts that can be accessed for breast cancer research. Some of these are listed in Supplemental Information S1. These studies vary by time, place, age, race/ethnicity, generation and availability of biospecimens. Many of these cohorts include women in age groups where mammograms are recommended and taken. This creates a highly cost-effective opportunity to discover prospective environmental predictors of MBD, particularly for cohorts with biospecimens taken during critical periods of breast development (in utero, puberty, pregnancy and postpartum, peri-menopause). Studies vary in timing of biospecimen collection, birth cohort, race/ethnicity, and geographic coverage within California. The Veterans Administration data present a further, unique opportunity to investigate MBD in women who served in various deployments where exposure to environmental toxins is expected or known. Statistical methods can standardize expected distributions of MBD based on age and BMI statistics for a given geographic area and expected MBD distributions can be compared to those observed. 

### 2.4. Beyond Descriptive Studies: ‘Omics

Once descriptive GIS and prospective epidemiological studies identify environmental chemical exposures that correlate with MBD for a given geographic area, an important next step is to identify individual biomarkers and susceptibility to complement the ecological data. This requires relevant biospecimens, either archived or contemporary. A range of ‘omics (e.g., genome, transcriptome, epigenome, metabolome) may be relevant and a significant advantage is offered by the metabolome [32]. Large cohorts from research studies with de novo data collection, including trials as well as cohorts designed from longitudinal medical records, provide important resources to examine MBD across a range of absolute risk based on genetic susceptibility. In addition to traditional gene–environment interaction studies, the metabolome is a read out of gene x environment interaction and provides a method of measuring phenotype that is closer to disease outcome [32]. Metabolomics offers the opportunity to describe biochemical pathways to risk. A stepped “meet in the middle approach” can be effective where environmental exposures are linked first to risk [33], in this case MBD, then to the metabolome. Simultaneously, MBD can be linked to the metabolome. The final step is to examine the pathway from environment to MBD via the metabolome. Clearly, the environment–MBD link is a critical first step that can be accomplished now using existing data. Moreover, recent experience with archived specimens supports the feasibility of metabolomics studies of WOS samples available in historic prospective cohorts [22,34,35]. Existing archived biological samples can be used to interrogate the individual responses to environmental exposures that distinguish women with higher MBD associated with environmental exposures.

### 2.5. Beyond Human Studies to Discover Mechanism

Integrating experimental toxicology with human environmental studies of MBD through supported, close collaboration will accelerate the discovery of causation and mechanism. This level of evidence strengthens advocacy and policy development for risk reduction and improved mammography screening efficiency. This step should not be left to chance. To accelerate discovery, close collaboration of human population scientists and experimental toxicologists is essential and requires incentives and monetary support. A recent example of how human and animal data can be interrogated together is found in metabolomic signatures of DDT in human specimens that were shown to be consistent with metabolomic signatures following controlled experimental DDT exposure in mice [22]. The insight of experimental toxicology will also facilitate targeted ‘omics using human specimens. Thus, the most effective collaborations would be iterative and long-term.

### 2.6. Creating Change to Prevent Breast Cancer

Change can be accelerated and is more likely to succeed when scientists partner with the communities they study and serve [36,37,38]. Both the GIS and cohort approach to finding environmental causes of MBD create opportunities for Community-Based Participatory Research (CBPR). GIS-based research can help to support and develop place-based advocacy for change that will likely be more effective and faster than slow diffusion of knowledge. Cohorts are also communities and participants can become CBPR partners, as demonstrated by the success of CBCR in the multigenerational Child Health and Development Studies multigenerational cohort [28,29]. 

Community partners working with the state and population scientists can also develop scalable and sustainable biomonitoring projects. A strong existing biomonitoring program in California [39] will facilitate quick adoption of community–scientist–state partnerships for breast-cancer-relevant bio-monitoring discovered via research on environmental correlates of MBD. 

## 3. Barriers and Solutions 

The problem of mixture exposures, including the overlap of environmental exposures, social and economic disparities and related psychosocial stress, can be addressed by querying multiple data bases to discover links. In a series of recent papers, Krieger and colleagues argue eloquently for using geospatial measures of “social polarization”, termed Indexes of Concentration at the Extremes (ICEs) [4,27,31]. These ICEs map social inequality that is linked to adverse health outcomes. Using ICE measures based on three critical components of social polarization: race/ethnicity, income or education and/or combined (e.g., ICE, based on race/ethnicity combined with income) provides a rich opportunity for prevention. Finding spatial correlates of risk can lead to community- and policy-level interventions to prevent their consequences. Krieger and colleagues have shown that ICEs can be better predictors of adverse environmental exposures (e.g., carcinogen exposure) than more traditional individual or neighborhood measures of socioeconomic position (SEP), such as individual income or neighborhood poverty level. New techniques of high-throughput metabolomics can discover pathways to MBD via environmental toxicants and individual responses to these toxicants, including identification of shared pathways that simplify the measurement of predictors. For example, a number of legacy environmental chemicals share metabolic pathways which might be investigated to find women at greatest risk [35]. 

Geographic cluster investigations of breast cancer cases present known methodologic problems due to low frequency of breast cancer in small cluster areas and high statistical uncertainty [40]. Importantly, monitoring a cancer biomarker that can be scored for many women in a geographic area addresses some of these limitations [40]. MBD is an excellent biomarker whose distribution can now be monitored in states where MBD is being routinely reported in medical records. Now, for the first time, we can monitor the geographic distribution of MBD, a strong surrogate for risk of breast cancer. 

As noted above, we expect exposures during WOS (in utero, during puberty, in pregnancy and early post-partum and during the peri-menopause) to have the strongest impacts on MBD. Discovery of the relation of environmental exposures during WOS to future MBD is made possible by combining birth cohort MBD with historical exposure data via NATA monitoring and pesticide monitoring in California. However, given that MBD responds quickly to changes in hormone medication [9], we cannot rule out more rapid changes in MBD following changes in environmental exposures even after WOS periods. Having infrastructure in place for geographic monitoring of MBD will create an opportunity to evaluate response to environmental events (e.g., chemical spills) or policy changes (e.g., changes in clean air protections) over time. MBD monitoring will also make it possible to help predict future breast cancer risk at the population level. 

Prospective epidemiological studies with available biospecimens or GIS data linked to environmental exposures and place of residence during WOS periods are an additional, complementary source of information on the etiology of MBD. Understanding the etiology of high MBD offers the opportunity for breast cancer prevention.

Until we query the geographic distribution of MBD, it is difficult to predict trends over place or time. Observing these patterns will determine the role of monitoring MBD and associated exposures as a tool for finding high-risk geographic areas and designing intervention policies and programs that can reduce risk of breast cancer. Initially, MBD monitoring is, appropriately, the subject of sound scientific research to establish feasibility and potential as a biomonitoring tool directed towards breast cancer prevention. If MBD monitoring is effective and if MBD is significantly tied to environmental factors, there are a number of state and federal agencies or community partnerships that may add MBD as a health indicator by place and time. Examples include The California Healthy Places Index [41], California State Department of Public Health, County Health Status Profiles [40] and the California Pan-Ethnic Health Network [42]. MBD monitoring, in conjunction with education and action by the strong and diverse breast cancer advocacy community in California, is also an additional opportunity that will be created by discovering geographic MBD patterns linked to environmental monitoring, or, alternatively, birth cohort MBD patterns linked to WOS exposure earlier in life.

## 4. Conclusions 

We propose an approach to accelerate etiological studies, as well as future biomonitoring of environmental exposure effects at the population level. Specifically, by using the opportunities available through clinical reporting as well as state-wide environmental data, we can efficiently examine whether environmental exposures are linked to MBD and inform strategies to reduce these exposures. These population-based strategies have advantages over any one single epidemiological study, which may have its own issues of selection bias. Thus, pairing registry data with individual cohort data will be a robust way to test hypotheses regarding environmental exposures and changes at the breast tissue level. 

It is highly likely that MBD and breast cancer share common determinants because of the strength of MBD associations with breast cancer. Environmental pressures on breast structure during WOS have been least studied and are a “black box”. We can look into this box using study designs that link exposures during WOS to MBD patterns over space and time using existing resources now in California. These designs fall in two categories: 1) Prospective studies of diverse cohorts with relevant biospecimens and/or residency history during susceptible periods in the life course, that can be linked to geographic data bases documenting environmental chemical exposure; 2) Ecologic studies linking residence patterns to MBD in communities. Follow-up studies can use the new and promising methods of high-throughput sensitive metabolomics that measure both toxics and individual responses in biospecimens that represent the result of gene by environmental interaction. Affiliated experimental toxicology studies with doses relevant to humans can increase certainty about causation and find mechanisms. These etiological studies are important when learning how to prevent breast cancer. Moreover, describing the geographic distribution of MBD can find communities at highest risk and environmental correlates of high risk. This information is critical to find vulnerable communities and create public policy to prevent breast cancer specific to place, as well as other known socioeconomic disparities in breast cancer risk that are strongly linked to place. For example, the recent national study conducted by White and colleagues provides evidence that supports clean air initiatives for breast cancer prevention [15]. Similarly, the discovery of disparate frequency of MBD by smaller geographic areas could create community advocacy for change. A very recent example of how MBD monitoring could create an opportunity for prevention is illustrated by a winning proposal by Nancy Buermeyer, Senior Policy Strategist, Breast Cancer Prevention Partners in the California Breast Cancer Research Program Global Challenge [26]. Her idea titled “California Ports: Air Pollution Interventions and Breast Cancer Risk in Local Communities” argued that residence near ports in California is accompanied by diesel exhaust exposure, which can easily be mitigated by reducing engine idling [39]. One major critique concerned how to evaluate the impact of a port intervention on breast cancer risk. MBD could be one mechanism for evaluation that avoids the low power of cancer case counts as an evaluation tool. 

Creating an initial model for MBD monitoring in relation to environmental exposures will open opportunities for breast cancer prevention that have not existed previously. Significantly, this approach uses existing data, making this approach feasible in the short-term and cost-effective. Mandated reporting of BI-RADS scores, in combination with existing cohort and environmental monitoring data by place and time, are available in California. MBD monitoring, in concert with environmental monitoring, is potentially scalable to other states with mandatory MBD reporting, available environmental data by place, and existing long-term cohorts with residence history and/or biospecimens during critical WOS.

## Supplementary Materials

The following are available online at https://www.mdpi.com/1660-4601/16/23/4731/s1, Information S1: Selected Population Studies for Finding Environmental Correlates of MBD.
