# Supplementary material for: Environmental Influences on Mammographic Breast Density in California: A Strategy to Reduce Breast Cancer Risk
Source: Int J Environ Res Public Health. 2019 Nov 27;16(23):4731. doi: 10.3390/ijerph16234731 (PMC6926682; doi:10.3390/ijerph16234731)
Supplement: Supplementary file 1 [file ijerph-16-04731-s001.pdf]

International Journal of
Environmental Research and Public Health

MDPI

Supplemental Information S1

Selected* Population Studies for Finding Environmental Correlates of MBD

1. California Studies

San Francisco Mammography Registry

https://mammography.ucsf.edu/

Rancho Bernardo Study

https://knit.ucsd.edu/ranchobernardostudy/study-description/

Multiethnic Cohort Study

https://www.uhcancercenter.org/mec

Child Health and Development Studies

http://www.chdstudies.org/

California Teachers Study

https://www.calteachersstudy.org/

Kaiser Permanente Division of Research (Northern California)

https://divisionofresearch.kaiserpermanente.org/

Kaiser Permanente Department of Research and Evaluation (Southern California)

https://www.kp-scalresearch.org/

CalVet 2013 Women Veterans Study

https://www.vacsp.research.va.gov/CSPEC/Studies/INVESTD-R/CalVet-2013-Women-Veterans-Study.asp

2. National Studies that may also have California participants with available MBD scores

Women's Health Initiative https://www.whi.org/SitePages/WHI%20Home.aspx

Nurses Health Study https://www.nurseshealthstudy.org/

Black Women's Health Study

https://epi.grants.cancer.gov/Consortia/members/blackwomen.html

Study of Women's Health Across the Nation (Swan) https://www.swanstudy.org/

*This list is not meant to be exhaustive. The reader may be aware of other opportunities in addition to these.
